# How to run a sustainability science research group sustainably?

**DOI:** 10.1007/s11625-020-00857-z

**Published:** 2020-08-25

**Authors:** Tobias Plieninger, Nora Fagerholm, Claudia Bieling

**Affiliations:** 1 Faculty of Organic Agricultural Sciences, University of Kassel, 34109 Kassel, Germany; 2 Department of Agricultural Economics and Rural Development, Georg-August-Universität Göttingen, Platz der Göttinger Sieben 5, 37073 Göttingen, Germany; 3 Department of Geography and Geology, University of Turku, 20014 Turku, Finland; 4 Division of Societal Transition and Agriculture (430b), University of Hohenheim, Schloss, 70593 Stuttgart, Germany

**Keywords:** Advocacy, Eco-anxiety, Research lab, Social tipping elements, Sustainability education, Sustainability transformations

## Abstract

Rigorous sustainability science includes addressing pressing real-world problems, weaving multiple knowledge systems, and striving for transformative change. However, these key attributes of sustainability science often conflict with university structures and established academic work practices, for instance with regard to frequent long-distance travel. Such contradictions between key principles of sustainability and everyday practices are experienced by many researchers not only at university level, but also in their individual behaviors. To help resolve this widespread divergence, we present ten principles to foster the sustainability of a research group working in sustainability science, based on our personal experiences and experiments as research group leaders. These principles comprise: (1) monitor the environmental footprint, (2) foster learning and innovation, (3) reduce the environmental footprint, (4) nurture campus sustainability, (5) embrace sustainability in private life, (6) constructively deal with environmental anxiety, (7) design research projects for sustainability impact, (8) engage with stakeholders, (9) capitalize on sustainability teaching, and (10) recognize biases and limits. Applying sustainability principles in everyday research practices can provide important social tipping points that may trigger the spreading of new social norms and behaviors.

## Introduction

Sustainability science has grown rapidly over the past 20 years. Being considered “not yet an autonomous field or discipline” in the early 2000s (Clark and Dickson 2003, p. 8060), sustainability science has now come of age for a while, as demonstrated by numerous journals, conferences, professorships, university departments and faculties, and research programmes (Spangenberg 2011). To guide society toward sustainability is a most central characteristic of sustainability science (Horcea-Milcu et al. 2019). Correspondingly, identifying, conceptualizing, and supporting seeds (Raudsepp-Hearne et al. 2019), leverage points (Abson et al. 2017), scenarios (Kishita et al. 2016), visions (Wiek and Iwaniec 2014), and pathways (Luederitz et al. 2017) towards sustainable development are overarching features of the field (Miller et al. 2014).

Rigorous sustainability science comprises, among other attributes, addressing pressing real-world problems (Schmidt and Pröpper 2017), weaving multiple knowledge systems (Tengö et al. 2017), and striving for transformative change of society globally (Díaz et al. 2019). However, these key attributes of sustainability science often conflict with university structures and established academic work practices (Haider et al. 2018). For example, flying to international conferences across the world remains a widespread (and sometimes required) practice in academia, food offered in university cafeterias is often unhealthy and not produced sustainably, and university endowments are frequently invested in fossil fuel companies (though these phenomena are increasingly questioned).

Many researchers experience these contradictions between key principles of sustainability and everyday practices not only at university level, but also in their individual behaviors (for instance, when flying back from academic meetings to have more time with their families). Such knowledge-action gap can inhibit impactful sustainability science in multiple ways. In particular, researchers can be affected by psychological stress, comprising negative mental and emotional consequences and leading to unclear role expectations and ultimately environmental anxiety (Usher et al. 2019). Also, contradictions between research outcomes and research practices may undermine credibility of sustainability scientists in the public (Fiske and Dupree 2014). Surprisingly, sustainability researchers and academics in ‘green’ research areas do not produce significantly less carbon emissions than their ‘non-green’ counterpart researchers (Wynes et al. 2019). Many sustainability scientists cope with this cognitive dissonance by suppressing inconsistencies and using justifications such as denial of control or responsibility, comparisons with the behavior of less-environmentally aware persons, and compensation through perceived sustainability benefits of their work (Schrems and Upham 2020).

**Fig. 1:**
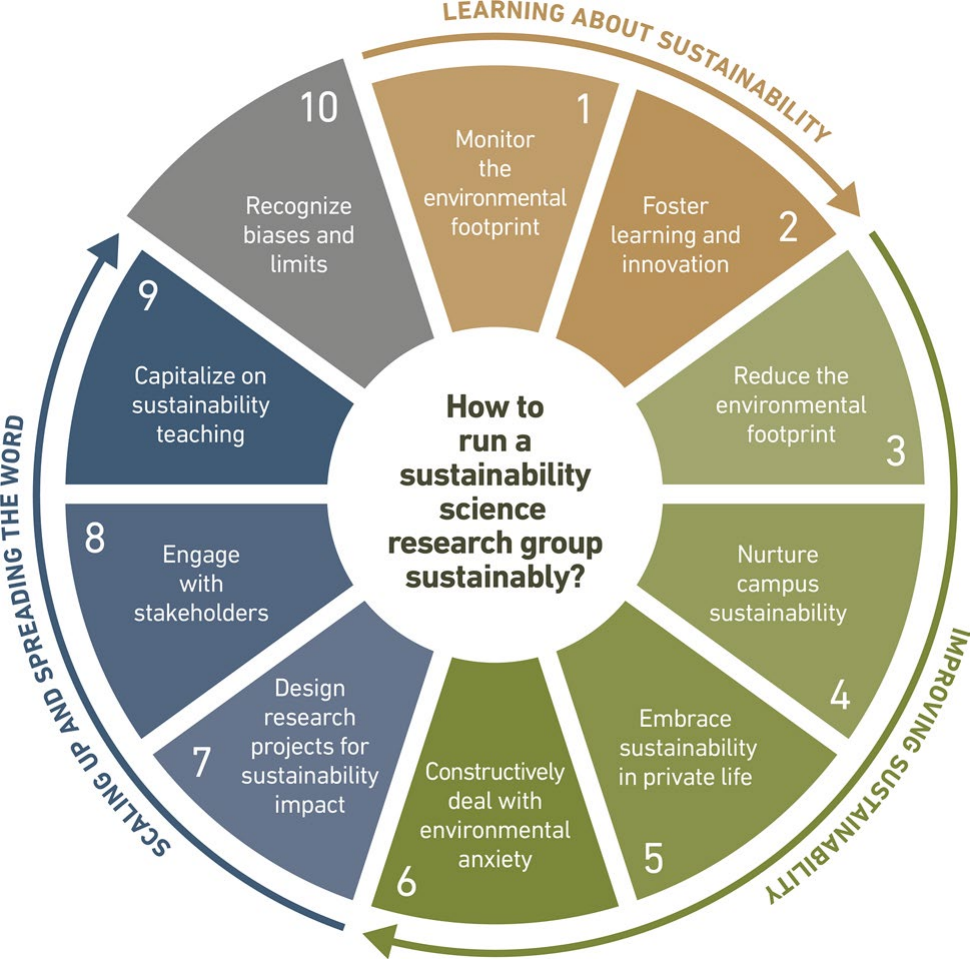
Set of ten principles for research groups in sustainability science for learning about sustainability, improving sustainability, and scaling up and spreading the word

The central argument of this note is that research groups are key drivers for shaping and implementing more reflective and more sustainable behaviors and by that for resolving the widespread knowledge-action gap in sustainability science. Therefore, they should receive more attention as nuclei for developing and living innovative sustainability practices. Our hope is that best practices implemented in leading sustainability science groups would spread to other groups and be upscaled to other disciplines eventually. We here suggest ten principles to foster the environmental sustainability of research groups working in sustainability science, based on our personal experiences and experiments as research group leaders at European universities. Our principles (Fig. 1) are grouped into clusters on ‘learning about sustainability’ (1, 2), ‘improving sustainability’ (3, 4, 5, 6), and ‘scaling up and spreading the word’ (7, 8, 9), while the final principle (10) is an overarching one.

## (1) Monitor the environmental footprint

To run a sustainability science research group in a sustainable manner starts by creating an understanding of the social-ecological impacts of different types of group activities. Build a knowledge basis to understand where the group currently stands, define common sustainability targets, and monitor impacts regularly, for example on an annual basis. Typically, the largest share of researchers’ environmental footprint is caused by professional travel, in particular when carried out by airplane and car (Kalmus 2019). Hence, the first step is to calculate the carbon footprint of the group’s travel activities. Several tools exist to perform this, but one recently developed calculator is targeted particularly to the scientific community: the travel carbon footprint calculator combines seven publicly available flight emission calculators and also considers train emissions (Barret 2020). It is specifically applicable to calculate the carbon footprint of a large set of trips and can help to identify a meeting place that minimizes emissions. A second step is to pay attention to energy and resource consumption, for example in relation to the use of internet (most notably cloud services), technical equipment, physical printing, and food catering. Although exact monitoring of energy and resource use may be impossible, current practices can be listed: what are the internet habits of the group, what cloud services are used, and where is big data analysis done? How energy-efficient are equipment, instruments, and processes being used? To what degree are they being shared? What activities produce the bulk of lab waste?

## (2) Foster learning and innovation

The relation between innovation and sustainability has many facets. On one hand, it is clear that we need to adopt new practices to become more sustainable and that technologies can greatly support us in this. On the other hand, particularly technological innovation can be even detrimental to sustainability when it leads to rebound effects. It might for instance make a lot of sense to continue to use and repair old printers, even though they are less energy-efficient than a new one promoted as ‘green’ and ‘sustainable’. Therefore, it is indispensable to critically assess available options (office infrastructure, networking procedures, etc.) with regard to their actual sustainability impacts and figure out what kind of innovation and learning is needed to become more sustainable. Using approaches such as social innovation (Mehmood et al. 2020) or design thinking (Fischer 2015) may offer opportunities to use the knowledge and skills of the research group members as catalysts to co-design innovative sustainability solutions.

Motivation towards sustainability also comes from following how colleagues perform in this task and from mutual learning. Such benchmarking between sustainability science research groups and wider academia can lead to good practices that can be adopted among a research group. Learning can be fostered by actively exchanging thoughts with colleagues at other institutions or by following arenas promoting discussion, such as blogs (e.g., Academic Flying 2020) or podcasts (e.g., ESSN 2020). The Covid-19 pandemic forced academia to remote working modes and pushed us seriously to master various technical platforms for teaching and collaboration. This disruption and the resulting digital leap have created new norms and innovations for working in an unexpectedly rapid manner. After the crisis, it will be essential to consider which of such practices to maintain instead of returning to practices that were more energy- or resource-intensive.

## (3) Reduce the environmental footprint

Although frequent air travel is not a precondition for success in academia (Wynes et al. 2019), long-distance travel to conferences, workshops, seminars, and field sites is common in sustainability science. Several steps can be taken to reduce this footprint. At least in Europe, prioritizing railway for mid-range academic travel is feasible. For example, thousands of academics in Germany have pledged not to use airplanes for trips of less than 1000 km distance. Innovative conference styles have potential for substantially lowering the environmental footprint of a conference while keeping the character of a live meeting and lowering barriers to inclusiveness. Templates are offered by the ‘Nearly Carbon-Neutral’ (NCN) conference (Hiltner 2020) or the ‘All continents, Balanced gender, low Carbon transport, Diverse backgrounds’ (ABCD) conference that mixes live-streamed and pre-recorded talks with in-person ones (Blackman et al. 2020). Changing annual live meetings to biannual ones may be another option. Such actions can reduce conference travel emissions by up to 90% (Klöwer et al. 2020). Department seminars and PhD defenses can also be moved into online formats, making them accessible to a much larger and wider audience. Smaller meetings, such as research group events, faculty search panels, and other committees, can take place in the virtual space as well.

In selection processes, committees should remove the number of physical talks given at distant departments from the evaluation criteria. Rather, visibility and networks can also be developed through original use of social media, engagement in academic societies, or editorial tasks. Due to their generally higher environmental footprints, senior academics bear more responsibility to reduce their footprints than junior ones. Reducing the environmental footprint of travel to field sites is more difficult: Does it make sense to ‘regionalize’ empirical sustainability research? To what degree can online surveys and remote sensing replace fieldwork on the ground? Can lesser and longer field campaigns replace more frequent and shorter travel to field sites? Can stronger reliance on local partners reduce travel?

Offsetting air travel-related carbon emissions by voluntary compensation schemes (now practiced for instance by some universities in Germany) is contested, but the purchase of serious gold standard certificates for unavoidable flight travel is in our view substantially better than flying without compensation. Changes in energy use have also potential to reduce a research group’s environmental footprint, for example by deploying conscious internet search engines (e.g., Ecosia), taking energy- and resources-aware decisions when purchasing lab equipment, or choosing a service provider that openly informs about energy demands.

## (4) Nurture campus sustainability

Universities are large institutions with high societal impact: They involve diverse actors with connections to numerous societal groups, constitute important economic players not only at local level, and provide key impulses through research and their ‘think tank’ character particularly in regard to economic and technological development. Moreover, they have a high reputation and serve as points of reference and orientation for the public and political decision-makers alike. More and more universities around the globe are currently setting examples by withdrawing investments in fossil fuel-based companies and suspending research that is connected to these. But typically, university administrations will only get active for sustainability when they perceive pressure to do so. Often, a few key persons have been enough to kick off university actions for sustainability. Universities can also nicely link research, teaching, and sustainability impact. For example, University of Natural Resources and Life Sciences (BOKU) Vienna (2020) established as the first university worldwide its own carbon offsetting scheme with projects in several countries. Off-setting academic travel of employees, but also critically assessing the social and ecological complexities of such off-setting in research and teaching is at the heart of this endeavor. A similarly participatory project has been developed with the ‘Stay grounded—keep connected’ strategy of ETH Zurich (2020). Other important tasks for universities to advance sustainability are enhancing energy and water resource efficiency of university campuses, upscaling the generation and use of renewable energy, establishing more sustainable university infrastructure development, and climate- and biodiversity-proofing of university greenspace management.

## (5) Embrace sustainability in private life

Many academics have fluidity in terms of separation of work and private lives. Although everybody has a right to a private life, a large discrepancy in sustainability goals and actions at work compared to those taken in one’s private life can jeopardize credibility (Attari et al. 2016). Being a sustainability scholar means we can spread the message not only at work. We also personally contribute to important social tipping points that may trigger the spreading of new social norms and individual behaviors (Centola et al. 2018). The ‘Value Belief Norm’ theory (Stern 2000), a theory of environmental behavior change, suggests that awareness of environmental consequences comes together with responsibility for these consequences. This fosters the personal norm of pro-environmental behavior, both in public and private lives. More importantly, pro-environmental behavior can be linked to wider co-benefits to one’s personal health and thus further motivate embracing sustainability (Cohen and Kantenbacher 2020). One everyday example is active transportation to work by foot or bicycle with substantial improvements to both personal health and climate indicators. The environmental stewardship literature offers rich examples of the linkages between consciousness, individual and collective environmental action, and personal well-being (Bennett et al. 2018).

## (6) Constructively deal with environmental anxiety

There are multiple ways of reacting to the current sustainability challenges. Given the existential and complex character of the issue, particularly young people struggle with feeling overwhelmed and intimidated (Taylor 2020). This may set free energy for taking action, but for many, at least at times, causes rather the contrary: they fall into despair, feel depressed, or adopt a fatalistic or cynical attitude (Usher et al. 2019). It is therefore an important part of a research group’s sustainability agenda to create an atmosphere and space in which people can openly exchange on their mental reactions to the current ecological and social crises. Considering ‘eco-anxiety’ as a normal reaction is a good first step towards taking it up constructively (Lawton 2019) and will relief already some pressure. A next step focuses on the development of positive visions of our future and pathways towards them, acting as shared counter-narratives to images of inescapable destruction and despair that are dominating in the current debate. Examples such as thousands of landscape-level sustainability initiatives (Carmenta et al. 2020), ‘bright spots’ (Cinner et al. 2016), or ‘seeds of good anthropocenes’ (Raudsepp-Hearne et al. 2019) help us to develop hope and imagine positive futures of a sustainable and good life as something achievable, although it will not come without backlashes and frustrations.

## (7) Design research projects for sustainability impact

While striving to minimize negative environmental impacts, we want to maximize the positive sustainability impact of our research. There are many directions for achieving impact, whether instrumental (triggering changes in practice and policy), conceptual (fostering new understanding), capacity building (training relevant actors), attitudinal/cultural (influencing societal values), or enduring connectivity (fostering follow-on interactions) impacts (Reed 2016). Societal impact can unfold at many spatial scales, from local to global ones, and can often be achieved by very simple means—although a more comprehensive design of projects for sustainability impact may require acquisition of some new skills.

One example are the self-made ‘innovation leaflets’ produced in the AGFORWARD project (2020) that proved very influential in upscaling novel agroforestry systems across Europe. Another possibility is to build impact activities into our formal research plans. For example, PhD researchers could be expected to develop at least one impact activity besides the typically three scholarly papers that comprise a dissertation. A next step is writing of a guidance document and best practices collection of the impact activities that are suitable for PhD projects. In such contexts, multiple synergies between academic and real-world impact can be created, for example when policy papers are published in scientific journals (see e.g., Pe'er et al. 2020). But beyond that, we consider it important to make striving for real-world impact an everyday practice. Potential opportunities include, among others, engaging local media when carrying out fieldwork; releasing targeted plain-language summaries for any research paper published; and creating and disseminating short video documentaries (see e.g., UTU Tanzania Team 2020). Helpful resources on how to achieve short- and long-term sustainability impacts are Reed (2016) and CommsConsult (2020).

## (8) Engage with stakeholders

Sustainability science has potential to spread the word to diverse groups of stakeholders at the science-society and the science-policy interfaces. A robust knowledge of the interests and needs of the stakeholders that are dependent on and/or influential for one’s research is key so that messages can be strategically targeted toward these actors (Kusmanoff et al. 2020). Multiple tools—partly simple, partly sophisticated—exist to identify, group, and understand stakeholders and the relationships among them (Reed et al. 2009). The most straightforward way to engage with stakeholders is integrating them as partners into research projects. We experienced stakeholder organisations such as the European Landowners’ Organisation (ELO), the International Union for Conservation of Nature (IUCN), or the World Business Council for Sustainable Development (WBCSD) as highly effective partners in European research projects. At more local levels, municipalities, regional businesses, associations, and even individuals are important stakeholders to ensure links to practice. The Intergovernmental Science-Policy Platform on Biodiversity and Ecosystem Services (IPBES) developed a format for stakeholder participation that can be adapted to multiple domains and levels of governance. Stakeholder engagement within IPBES has helped to communicate, disseminate, and implement findings; to co-develop guidelines and measures for biodiversity conservation within member countries; and to create linkages between global policy and local actors (Krug et al. 2020). Similarly, the BiodivERsA platform offers detailed resources for informing and engaging with stakeholders (Durham et al. 2014). At best, stakeholder engagement leads to knowledge co-creation and supports transformative changes to sustainability.

## (9) Capitalize on sustainability teaching

While many researchers aim for creating leverage for sustainability through research projects and publications, teaching is also a key arena and tool to achieve sustainability. In many countries throughout the world, research-based teaching is a key element of higher education, and correspondingly, a large share of researchers is in close contact with students as part of their daily work. This involves a huge potential: after finishing their study programs, generations of students pursue careers in relevant natural resource management agencies, NGOs, or companies at various levels and in diverse fields, thus becoming decision-makers in all societal spheres. Spreading the idea of sustainability and making it part of the worldview and commitment of students—not only in sustainability teaching, but in all kinds of classes—is therefore a very powerful, yet relatively easy to accomplish task for researchers. What is necessary for this is establishing a strong and clear link to the global sustainability agenda throughout all kinds of teaching offers, for example organized along the UN-Sustainable Development Goals (Bowser et al. 2020). For this, the manifold experiences, role models, and tools established within Education for Sustainable Development can be drawn upon (Mulà et al. 2017). An essential element is a focus on real-world problems, to be achieved for instance in the course of project work that considers the complexity of the matter, but also opens up pragmatic and solutions-oriented pathways for action. Innovative teaching formats for sustainable development, for instance using living labs to engage students with applied sustainability challenges (Evans et al. 2015), allow for handing over responsibility to students. This can create life-changing experiences to them and promote long-lasting impacts (Chawla 1998).

## (10) Recognize biases and limits

Although the potential for promoting sustainability in a research group is vast, there are limitations. In practical terms, any research activity comes with social-ecological trade-offs, ambiguities, and compromises, so that we have to accept that we cannot act 100% perfect in our lives. For some eminent workshops, conferences, or stakeholder contacts, long-distance air travel may simply be irreplaceable (and useful). While it may be rewarding to strengthen research activities close to campus, the Global South is known to be particularly rich in sustainability challenges and lessons (Nagendra 2018), and North–South exchange is a fundamental pillar of sustainability science (Kates et al. 2001).

In more fundamental terms, navigating normativity in sustainability science may pose challenges. Sustainability science is a value-laden or ‘crisis discipline’ (Chan 2008) and—in the interest of promoting sustainability—to some degree departs from ‘objective’ science in that it comprises a facts-based and a normative dimension. Most sustainability scientists embrace science-based advocacy for sustainable development (Shrivastava et al. 2020). However, how to (and how not to) advocate for sustainability in policy and practice is more contested. For getting the facts straight, it is indispensable to strictly consider good scientific practice codes and use rigorous peer-review processes. We can avoid misusing the scientific process also by grounding policy and practice recommendations firmly in our own scientific expertise. Peery et al. (2019) provide details on how to avoid activities outside scientific norms that we consider valid for sustainability science. For the normative dimension, we need to make our background and claims explicit and consider the ones of other people, to open up a debate on the many different things sustainability might mean in a specific context, on who should take over which responsibility, and on how to settle the manifold dilemmas and conflicts in sustainability-directed action. In very practical terms, building up a research group that comprises students and scientists of diverse backgrounds and values may be the best premise for delivering good sustainability science to society.

## Conclusions

In this note, we provide a set of principles for reducing the negative and increasing the positive impacts of a research group. Most principles focus on environmental sustainability, while we keep the multiple interactions between social and ecological sustainability dimensions in mind. The solutions that individual research groups and their leaders can contribute may appear small given the magnitude of current sustainability challenges. Many relevant decisions may not lie in the hands of a research group. Also, there is a debate on the effectiveness of individual action for sustainability (Marris 2020). However, we argue that reducing a sustainability scientist’s environmental footprint is setting an important example, with high potential for multiplication and scaling. This potential can be further increased by speaking up for sustainability in social media and the mass media and by identifying ‘allies’ in other disciplines and making them change agents. Addressing sustainability issues that researchers can influence through their own activities at the level of research groups can be particularly powerful by mediating between individual and—undoubtedly indispensable—collective and institution-level action for sustainability. Thus, applying sustainability principles in everyday research practices can provide important social tipping points that may trigger the spreading of new social norms and behaviors, but also policies and economic processes (Nyborg et al. 2016; Otto et al. 2020).
